# Expression of a novel mycobacterial phosphodiesterase successfully lowers cAMP levels resulting in reduced tolerance to cell wall–targeting antimicrobials

**DOI:** 10.1016/j.jbc.2022.102151

**Published:** 2022-06-17

**Authors:** Michael Thomson, Yi Liu, Kanokkan Nunta, Ashleigh Cheyne, Nadia Fernandes, Richard Williams, Acely Garza-Garcia, Gerald Larrouy-Maumus

**Affiliations:** 1 MRC Centre for Molecular Bacteriology and Infection, Department of Life Sciences, Faculty of Natural Sciences, Imperial College London, London, United Kingdom; 2 Imperial BRC Genomics Facility, Imperial College London, London, United Kingdom; 3 The Francis Crick Institute, London, United Kingdom

**Keywords:** mycobacteria, 3′,5′-cAMP, peptidoglycan, antimicrobials tolerance, bioenergetics, phosphodiesterases, AEC, adenylate energy charge, AMR, antimicrobial resistance, BCA, bicinchoninic acid, DCS, D-cycloserine, DiOC_2_, 3,3′ diethyloxicarbocianide chloride, ECAR, extracellular acidification rate, ETC, electron transport chain, *m*-DAP, meso-diaminopimelic acid, MIC_50_, minimal inhibitory concentrations, MS, mass spectrometry, MurNAc, *N*-acetylmuramic acid, OCR, oxygen consumption rate, PDE, phosphodiesterase, PG, peptidoglycan, SEC, size-exclusion chromatography, THDP, 2,3,4,5-tetrahydrodipicolinate

## Abstract

cAMP and antimicrobial susceptibility in mycobacteriaAntimicrobial tolerance, the ability to survive exposure to antimicrobials *via* transient nonspecific means, promotes the development of antimicrobial resistance (AMR). The study of the molecular mechanisms that result in antimicrobial tolerance is therefore essential for the understanding of AMR. In gram-negative bacteria, the second messenger molecule 3′’,5′’-cAMP has been previously shown to be involved in AMR. In mycobacteria, however, the role of cAMP in antimicrobial tolerance has been difficult to probe due to its particular complexity. In order to address this difficulty, here, through unbiased biochemical approaches consisting in the fractionation of clear protein lysate from a mycobacterial strain deleted for the known cAMP phosphodiesterase (Rv0805c) combined with mass spectrometry techniques, we identified a novel cyclic nucleotide-degrading phosphodiesterase enzyme (Rv1339) and developed a system to significantly decrease intracellular cAMP levels through plasmid expression of Rv1339 using the constitutive expression system, pVV16. In *Mycobacterium smegmatis* mc^2^155, we demonstrate that recombinant expression of Rv1339 reduced cAMP levels threefold and resulted in altered gene expression, impaired bioenergetics, and a disruption in peptidoglycan biosynthesis leading to decreased tolerance to antimicrobials that target cell wall synthesis such as ethambutol, D-cycloserine, and vancomycin. This work increases our understanding of the role of cAMP in mycobacterial antimicrobial tolerance, and our observations suggest that nucleotide signaling may represent a new target for the development of antimicrobial therapies.

Along with climate change and viral pandemics, antimicrobial resistance (AMR) is currently one of the greatest threats to human health. AMR is preceded by antimicrobial tolerance (1, 2, 3). Antimicrobial tolerance results from transient and reversible physiological adaptations that orchestrate the remodeling of bacterial physiology in order to reduce their susceptibility to antimicrobials (2, 3). The second messenger 3′,5′-cAMP has been implicated in AMR (4, 5, 6, 7, 8). In gram-negative bacteria, studies have linked cAMP signaling to antibiotic resistance (9, 10). Alper *et al.* demonstrated that strains of *Salmonella typhimurium* with mutations in key components of the cAMP signaling system display partial resistance to 20 commonly used antibiotics (9). Moreover, Kary *et al.* reported that *S. typhimurium* strains with altered levels of cAMP or lacking Crp, a cAMP-activated global transcriptional regulator, exhibit reduced susceptibility to fluoroquinolones as a result of decreased permeability and increased efflux (10).

The link between cAMP signaling and AMR and/or antimicrobial tolerance in mycobacteria, a genus that comprises both free-living species and important pathogens and that includes *Mycobacterium tuberculosis*, the vaccine strain *Mycobacterium bovis* BCG and the model organism *Mycobacterium smegmatis*, has not been thoroughly explored. This lack of knowledge is partially due to the complexity of the mycobacterial cAMP signaling system, as well as to the lack of effective tools to manipulate it (11, 12, 13, 14, 15). *Escherichia coli* has only one enzyme that generates cAMP, the adenylate cyclase Cya, and a single enzyme that hydrolyzes cAMP, the phosphodiesterase (PDE) CpdA(16). In contrast, the genome of the *M. tuberculosis* type strain H37Rv encodes 16 proteins predicted to have adenylate cyclase activity. Compared to *E. coli* Cya, which comprises one regulatory and one catalytic domain, mycobacterial adenylate cyclases can contain different regulatory domains, transmembrane regions, and even other enzymatic domains (15, 16). As for cAMP degradation, only one mycobacterial PDE, encoded by *rv0805*, has been reported. However, *rv0805* is a gene only found in *M. tuberculosis* and very closely related species (17, 18, 19, 20, 21), while cAMP PDE activity has been reported to be present in more distantly related species such as *M. smegmatis* (22), suggesting the existence of an additional PDE that is likely more ubiquitous in the mycobacterial genus. This expectation is supported by the fact that Rv0805 is more active toward 2′,3′-cAMP, which arises from RNA degradation, than toward the bona fide second messenger 3′,5′-cAMP (22, 24). The high number of cAMP-producing enzymes and the poor activity of the known PDE complicate the manipulation of the mycobacterial cAMP signaling and have crippled the investigation of its contribution to AMR and/or antimicrobial tolerance.

To tackle this challenge, we searched for a molecular tool that would allow us to efficiently decrease intracellular cAMP levels. The currently available exogenous strategies for altering cAMP levels, including the use of an adenylate cyclase activator (forskolin) and analogs such as dibutyryl-cAMP, only result in modest changes in intracellular cAMP levels (23, 24). Meanwhile, expression of the known PDE (Rv0805) in the laboratory strain *M. smegmatis* mc^2^155 leads to only a ∼0.2-fold decrease in cAMP levels (18). Instead, we focused on finding the “missing” conserved PDE using a combination of classical biochemical approaches and mass spectrometry (MS) techniques. With this strategy, we identified Rv1339, an uncharacterized cyclic nucleotide PDE that is ubiquitous in mycobacteria. Phylogenetic analyses showed that Rv1339 is a member of a poorly characterized group of PDE enzymes with a metallo-β-lactamase fold that is present in several bacterial phyla. This group is distinct from, but evolutionarily related to, the typical class-II PDEs, a small group of PDEs mainly found in unicellular eukaryotes (25).

Plasmid expression of Rv1339 in *M. smegmatis* mc^2^155 led to a threefold decrease in intracellular cAMP levels. By examining the bacterial transcriptome, metabolome, and bioenergetics, we observed that Rv1339 activity resulted in changes in gene expression, altered bioenergetics, and disrupted peptidoglycan (PG) biosynthesis, leading to a decrease in tolerance specific to antimicrobials that target cell wall assembly. These findings raise the possibility that cAMP signaling can be a promising new target for the development of compounds that inhibit antimicrobial tolerance in mycobacteria.

## Results and discussion

### Rv1339 is a cyclic nucleotide-specific, atypical class-II PDE

To identify novel sources of cyclic nucleotide PDE activity in mycobacteria, we used an unbiased biochemical approach involving the sequential fractionation of *M. bovis* BCG Δ*rv0805* lysate coupled to a TLC-based cAMP PDE activity assay (22, 26). The enrichment of cAMP PDE activity was achieved through two chromatography steps that separated proteins first according to their charge and then based on their molecular weight (Fig. S1). After the first purification step, the active fractions were identified and pooled before the second step. Subsequently, trypsin digestion and proteomic analysis were used to identify the proteins present in the second set of active fractions. Out of eight proteins that were uniquely present in the active fractions, three were uncharacterized proteins: Rv0250, Rv2568, and Rv1339. Saturating transposon mutagenesis has shown that *rv0250*, *rv2568*, and *rv1339* are all nonessential for growth *in vitro* (27). However, *rv1339* is necessary for persistence in the mouse model (28). Moreover, Rv1339 was annotated to have hydrolase activity and we found orthologs of the *rv1339* in most of the mycobacterial genomes we analyzed. We therefore decided to focus on the characterization of Rv1339.

**Figure 1:**
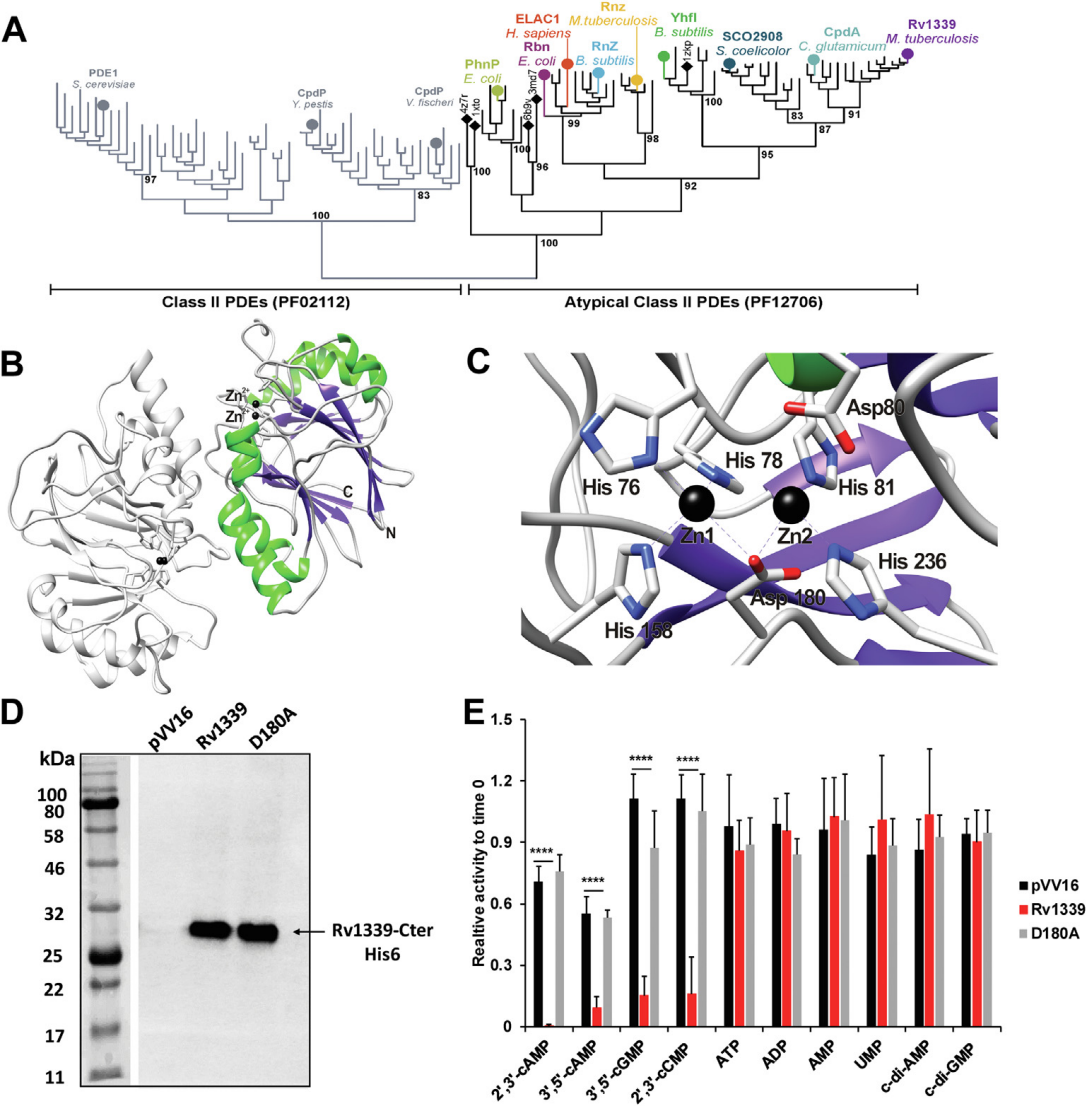
**Phylogenetic analysis, structure prediction, recombinant expression, and cell-free extract *in vitro* activity of Rv1339 expressed in *M. smegmatis* mc**^**2**^**155.***A*, maximum-likelihood phylogenetic consensus tree of selected members of typical (PF02112) and atypical (PF12706) phosphodiesterase (PDE) class-II families. *B*, ribbon representation of a homology model of Rv1339 established using Modeller 9.23 (115) with the crystal structure of the metallo-β-lactamase fold protein YhfI from *Bacillus subtilis* (PDB: 6KNS) as the template. YhfI was found to be a homodimer in solution by size-exclusion chromatography (29). The structure exhibits the alpha-beta-sandwich configuration characteristic of the metallo-β-lactamase fold, which consists of two β sheets of seven β strands each (*purple*) and α helices (*green*) capping the β sheet. The two zinc cations in the active sites are depicted in *black*. The figure was created using UCSF Chimaera (116). *C*, zoomed view of one of the active sites of the homology model of Rv1339 showing the coordinating histidine and aspartate residues. Asp180 was chosen for mutagenesis because it appears to be involved in the coordination of both zinc cations. *D*, Western blot of the pVV16 (empty vector) control, Rv1339-expressing, and Rv1339 D180-expressing *M. smegmatis* mc^2^155 strains. The proteins were probed with an α-His antibody, and each lane was loaded with 50 μg of clear soluble lysate. *E*, relative PDE activity of the clear soluble lysate after 30 min of incubation compared to time 0 for the pVV16 (empty vector) control (*black*), Rv1339-expressing (*red*) and Rv1339 D180-expressing (*gray*) *M. smegmatis* mc^2^155 strains. The data are presented as the means ± SDs of two biological replicates and three technical replicates. Unpaired two-tailed Student’s *t* tests were used to compare the data, and *p* < 0.05 was considered significant. ∗∗∗∗*p* < 0.0001 as analyzed by Student's *t* test. PDB, Protein Data Bank.

Literature mining and phylogenetic analyses revealed that Rv1339, YfhI from *Bacillus subtilis* (29), and CpdA from *Corynebacterium glutamicum* (30) are representatives of a previously unrecognized class of PDEs defined by the signature metal-binding motif [T/S]HXHXDH, where X tends to be a hydrophobic, small or very small residue. This motif is reminiscent of the bona fide class-II PDE motif HXHLDH(27), and indeed, the two families are evolutionarily related—belonging to the metallohydrolase/oxidoreductase superfamily (SSF56281). We propose to name this novel group atypical class-II PDEs. Interestingly, atypical class-II PDEs are closely related and might have evolved from the ribonuclease-Z family of proteins involved in the maturation of tRNA (Fig. 1*A*).

To generate a catalytically inactive form of Rv1339, we built a 3D structure homology model of Rv1339 to inform our site-directed mutagenesis efforts (Fig. 1*B*) (31). Based on this model, we chose to mutate to an alanine the aspartate residue at position 180 (D180A), as this residue appears to be involved in the binding of both divalent metal ions in the metal-binding site (Fig. 1*C*). An aspartate residue at this position has been described in the literature as being required for the activity of other metallo-β-lactamase proteins (32). Using a replicative plasmid under the control of the moderate constitutive promoter hsp60, the different constructs generated in this study were successfully expressed in *M. smegmatis* mc^2^155 (Fig. 1*D*).

To study the substrate specificity of Rv1339, we attempted to clone, express, and purify the enzyme. Unfortunately, despite the use of several different expression systems, our attempts to generate enough homogeneous purified enzyme were unsuccessful. We therefore resorted to measure nucleotide hydrolysis using cell-free extracts of the pVV16 (empty vector) control, Rv1339-expressing and Rv1339 D180A-expressing *M. smegmatis* mc^2^155 strains (Fig. 1*D*). Their relative activity in hydrolyzing the chosen nucleotides was measured by LC-MS after 30 min of incubation of each one of the nucleotides at a final concentration of 10 μM with 30 μg of cell-free extract. Values were compared to those of an aliquot taken at 0 min. Based on the literature (21, 33, 34, 35, 36, 37, 38, 39), we chose to test 10 different molecules (2′,3′-cAMP; 3′,5′-cAMP; 3′,5′-cGMP; 2′,3′-cCMP; ATP; ADP; AMP; UMP; c-di-AMP; and c-di-GMP). As seen in Figure 1*E*, after 30 min of incubation, the levels of 2′,3′-cAMP and 3′,5′-cAMP were barely detectable in the Rv1339-expressing strain, while these levels in the control strain transformed with the empty vector were decreased only by 40%, a decrease we attributed to the activity of endogenous PDEs. Importantly, the cAMP hydrolyzing activity of the Rv1339 D180A-expressing strain was comparable to that of the empty vector control, indicative that Rv1339 D180A is indeed catalytically inactive. A similar result was obtained for 3′,5′-cGMP and 2′,3′-cCMP. No catalytic activity was observed toward ATP, ADP, AMP, UMP, c-di-AMP, or c-di-GMP.

Our data show that Rv1339 is a cyclic nucleotide PDE with broad specificity, as has been reported for other cyclic nucleotide PDEs (21, 30, 40, 41, 42) and suggest that Rv1339 is a multifunctional PDE capable of hydrolyzing diverse cyclic nucleotides *in vivo*.

### The expression of Rv1339 leads to a decrease in intracellular cAMP levels and turnover, a growth defect and compromised bacterial bioenergetics

**Figure 2:**
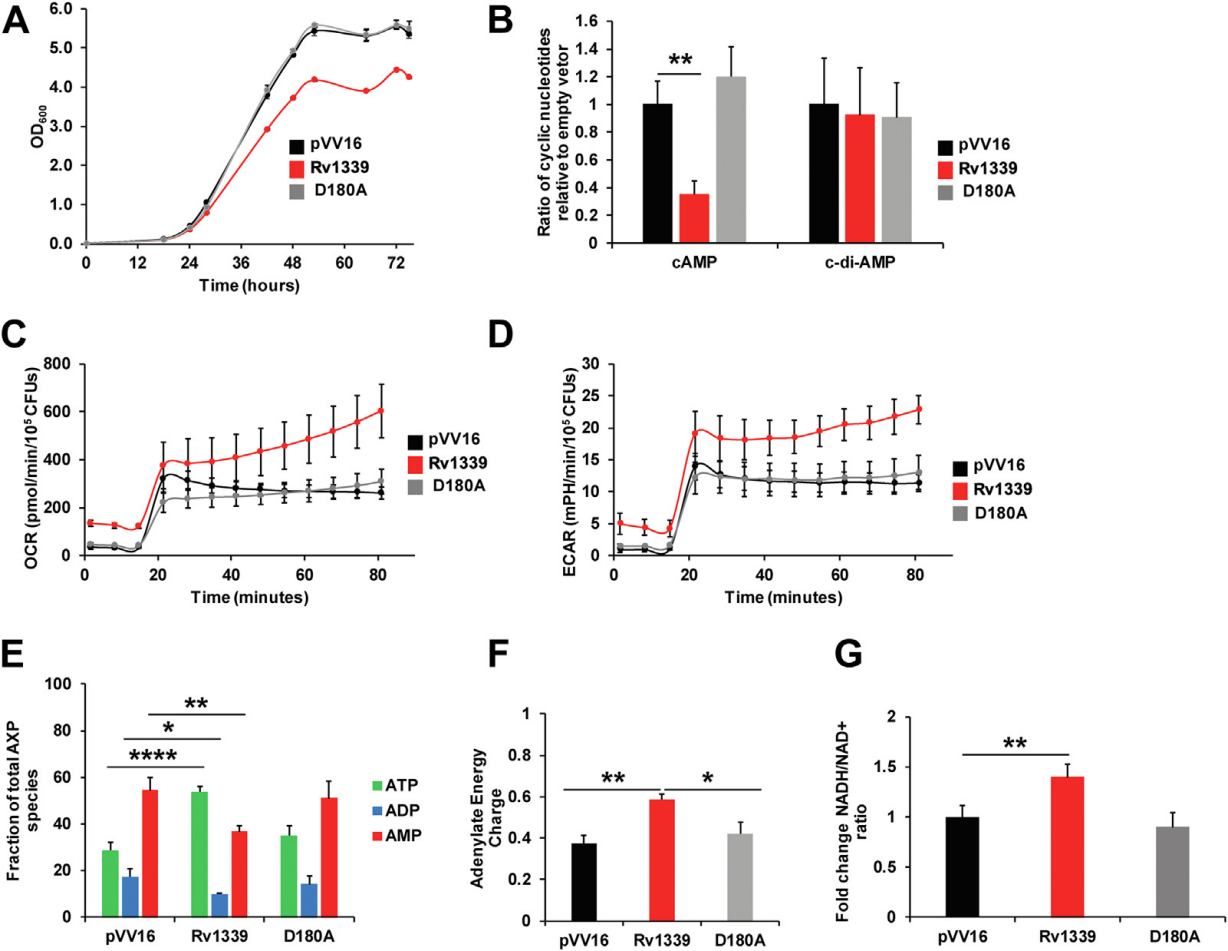
**Expression of Rv1339 in *M. smegmatis* mc**^**2**^**155 compromises growth, depletes cAMP, and redirects bioenergetics toward ATP synthesis.***A*, growth curves in 7H9 medium of the pVV16 (empty vector) control (*black*), Rv1339-expressing (*red*) and Rv1339 D180-expressing (*gray*) *M. smegmatis* mc^2^155 strains. *B*, intracellular cAMP and c-di-AMP levels in these strains measured by LC-MS. Oxygen consumption rates (*C*) and extracellular acidification rates (*D*) of the strains measured using Seahorse XFP analysis. The first three measurement cycles were obtained in the absence of a carbon source after which a mixture of glucose and glycerol was injected to a final concentration of 0.2% each. *E*, fractions of total AXP species in the mid-log phase of bacterial growth of the strains measured by LC-MS. *F*, calculated adenylate energy charge (AEC; (*F*)) and fold change in the NADH/NAD^+^ ratio measured using the alcohol dehydrogenase method (*G*). Increased AEC and NADH/NAD^+^ ratios in the Rv1339-expressing strain indicate a shift toward synthesis of ATP. The statistical analysis was performed with a two-tailed Student’s *t* test. ∗*p* < 0.05, ∗∗*p* < 0.01, ∗∗∗*p* < 0.001 and ∗∗∗∗*p* < 0.0001. The data are presented as the means ± SDs of three biological replicates and three replicates.

To investigate the potential *in vivo* PDE activity of Rv1339, we first monitored the growth of the *M. smegmatis* mc^2^155 empty vector control, Rv1339-expressing and Rv1339 D180A-expressing strains. The strain expressing Rv1339, but not the strain expressing Rv1339 D180A or the empty vector control, displayed a minor growth decrease of approximately 30% when cultured in 7H9 medium supplemented with glucose and glycerol carbon sources (Fig. 2*A* and Table S1). These results suggest that Rv1339 constitutive expression negatively affects bacterial physiology.

To determine whether the expression of Rv1339 in *M. smegmatis* mc^2^155 alters the levels of intracellular signaling nucleotides *in vivo*, we measured intracellular cAMP and c-di-AMP levels using LC-MS (43). In agreement with the *in vitro* results with cell-free extracts (Fig. 1*E*), the expression of Rv1339 led to a 3.2-fold decrease in the intracellular cAMP levels, but no decrease in the intracellular c-di-AMP levels was observed (Fig. 2*B*). We also measured the incorporation of ^13^C through [U-^13^C_6_]-glucose and [U-^13^C_3_]-glycerol stable isotope tracing and found a twofold increase in turnover of cAMP at 0.5 doubling time (Fig. S2*A*), suggesting that the Rv1339-expressing strain exhibits higher cAMP turnover than the empty vector control strain. These experiments show that when expressed in *M. smegmatis* mc^2^155, Rv1339 possesses cAMP PDE activity *in vivo*.

As cAMP is synthesized from ATP by the action of adenylate cyclases, we hypothesized that altered cAMP levels caused by the expression of Rv1339 would compromise *M. smegmatis* bioenergetics and alter the AXP (ATP, ADP, and AMP) pool. Therefore, a multipronged approach was used to investigate the effects of decreased intracellular cAMP levels on bacterial bioenergetics. Seahorse XFP analysis was used to determine the extracellular acidification rate (ECAR) and oxygen consumption rate (OCR) (44, 45, 46) of the empty vector control, Rv1339-expressing and Rv1339 D180A-expressing *M. smegmatis* mc^2^155 strains in the presence and absence of glycerol and glucose, which are the conventional carbon sources used for the growth of mycobacteria *in vitro*. ECAR is the readout of carbon catabolism and the tricarboxylic acid cycle due to the production of H^+^ as a result of glycolysis or NADH/H^+^ synthesis. NADH is a reducing equivalent/electron donor that feeds electrons into the menaquinone pool and then into the electron transport chain (ETC). The OCR is a readout of the activity of the ETC. A protocol was designed based on similar studies reported in the literature (45, 46). In order to gain an understanding of the basal bioenergetic capacity of the strains, the basal OCR and ECAR in the absence of a carbon source were measured after three cycles of measurements over the course of about 20 min (1 cycle = 4 min of mixing and 2 min of reading). The data showed that Rv1339-expressing bacteria exhibit a higher basal OCR and ECAR over the course of the three initial measurements (∼20 min), which indicates that the bioenergetic machinery was functioning at increased levels in the Rv1339-expressing strain, even in the absence of a carbon source (Fig. 2, *C* and *D*). After 20 min, glycerol and glucose were injected at a final concentration of 0.2%, to match the carbon concentration found in the bacterial culture medium used in all other experiments. Once the carbon sources were injected, all bacterial strains displayed increases of more than 10-fold in their OCR and ECAR. However, in the Rv1339-expressing bacteria, the levels of OCR and ECAR throughout the rest of the experimental period were significantly higher than those of the empty vector control strain and could be indicative of an increase in glycolytic activity. The strain expressing the inactive mutant Rv1339 D180A displayed a similar OCR and ECAR to the empty vector control strain throughout the assay. The differences between the empty vector control and the R1339 D180A-expressing strain were not statistically significant, indicating that changes in ECAR and OCR were brought about by Rv1339 in a catalytically dependent manner. To validate these results, we decided to measure the AXP pool and the NADH/NAD+ ratio as readouts of the ECAR and OCR.

Increased OCR and ECAR activity should be correlated with an altered AXP pool and therefore an increase in the adenylate energy charge (AEC). The AEC determines the energy state of the cell. AEC, defined by Atkinson and Walton (47) as the ratio of the mole fraction of ATP plus half the mole fraction of ADP in the total adenine nucleotide pool (ATP + 1/2 ADP/AMP + ADP + ATP), is widely used as a measure of the available energy stored in adenine nucleotides and, hence, as a parameter indicating the energy status and metabolic potential of cells (48, 49, 50). In mycobacteria, the AEC value ranges from 0.4 to 0.8 (51). More specifically, an increase in the AEC will promote an ATP-utilizing pathway. On the other hand, a decrease in the AEC will correspond to an ATP-generating pathway. Similarly, an increased ECAR should correlate with increased NADH levels because NADH is produced by carbon catabolism and TCA cycle activity (45). To confirm our hypothesis that the Rv1339-expressing strain would have a higher AEC, we measured AXP levels by LC-MS and calculated the AEC in the empty vector control, Rv1339-expressing and Rv1339 D180A-expressing *M. smegmatis* mc^2^155 strains (Fig. 2, *E* and *F*).

As shown in Figure 2*E*, under the culture conditions tested in this study, the empty vector control strain displayed a total AXP fraction consisting of ∼30% ATP, ∼20% ADP, and ∼50% AMP. However, in the Rv1339-expressing *M. smegmatis* mc^2^155 strain, the total AXP fraction consisted of ∼50% ATP, ∼10% ADP, and ∼40% AMP. Furthermore, in the Rv1339 D180A-expressing *M. smegmatis* mc^2^155 strain, the total AXP fraction was similar to that in the empty control strain. These ratios result in a 50% increase in the AEC value, from ∼0.4 in the empty vector control strain and Rv1339 D180A-expressing strain to ∼0.6 in the Rv1339-expressing strain (Fig. 2*F*). These data confirm that the AEC is shifted toward an ATP-utilizing pathway, which can be a result of the need to redirect the pool of AXP toward ATP synthesis to compensate for the depletion of cAMP due to Rv1339 PDE activity.

In addition, as mentioned earlier, an increase in ECAR should correlate to an increase in NADH/NAD+ ratio as a result of increased glycolysis, and NADH would feed the ETC to sustain the production of ATP. In order to confirm this prediction, we decided to measure the NADH/NAD+ ratio. The NADH/NAD^+^ ratio was increased by 0.5-fold in the Rv1339-expressing strain relative to the empty vector control (*p* < 0.01) and Rv1339 D180A-expressing strain (Fig. 2*G*), which was indicative of an increase in carbon catabolism and confirmed the Seahorse measurements (Fig. 2*D*). To further validate the changes in carbon catabolism activities, the turnover of serine, which can be used as a readout of glycolytic activity (52), was measured by [U-^13^C_6_]-glucose and [U-^13^C_3_]-glycerol stable isotope labeling (Fig. S2*B*). This analysis clearly showed that Rv1339-expressing bacteria exhibited increased ^13^C incorporation (*e.g.*, m + 3 of serine was increased by 10% in the Rv1339 strain relative to the empty vector control strain) (Fig. S2*B* and Table S5). This finding indicates an increased turnover of serine and, thus, increased glycolytic activity.

Taken together, these results show that the expression of Rv1339 leads to a decrease in cAMP levels, which in turn redirects bioenergetics towards ATP production *via* increased glycolysis and AEC.

### The expression of Rv1339 alters the electron transport chain and PG biosynthesis

To further investigate the mechanisms underlying phenotypic and bioenergetic remodeling in the Rv1339-expressing *M. smegmatis* mc^2^155 strain, we conducted RNA sequencing, microbiological assays, and untargeted metabolomic analyses.

**Figure 3:**
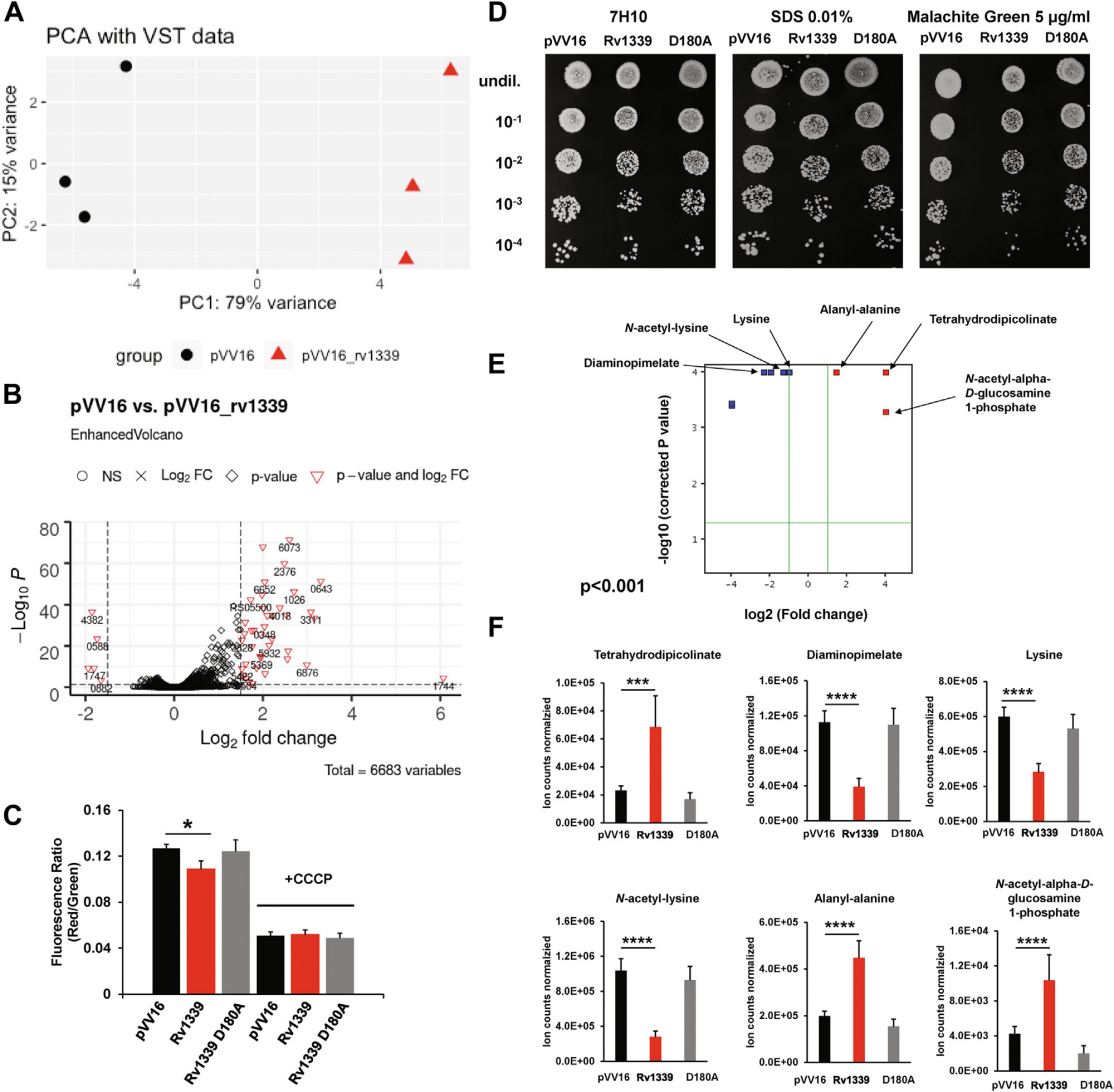
**Expression of Rv1339 alters the transcriptome, compromises cell wall integrity, and disrupts peptidoglycan biosynthesis.***A*, RNA-Seq of the pVV16 (empty vector) control and Rv1339-expressing *M. smegmatis* mc^2^155 strains in the mid-log phase of growth. Principal component analysis (PCA) assessing the quality of data produced using normalized counts subjected to variance stabilizing transformation (VST). Technical replicates appear to cluster in accordance with their respective experimental group. *B*, volcano plot illustrating log2 fold changes and adjusted *p*-values of each gene in the differential gene expression analysis of Rv1339-expressing strain compared with the empty control vector strain. *Horizontal dotted line* shows adjusted *p*-value cut-off at 0.05 and *vertical dotted* lines show log2 fold change cut-off at a magnitude of 1.5. Nonsignificantly differentially expressed genes with log2 fold change under 1.5 magnitude are indicated by a *black circle*; nonsignificantly differentially expressed genes with log2 fold change over 1.5 magnitude are indicated by a *black cross*; significantly differentially expressed genes with log2 fold change under 1.5 magnitude are indicated by a *black diamond*; and significantly differentially expressed genes with log2 fold change over 1.5 magnitude are indicated by *red triangles* and labeled with their old locus tags (where this tag was unavailable, respective new locus tags were used). *C*, membrane potential, expressed as fluorescence ratio (Red/Green) of DiOC2, of intact cells and cells treated with the depolarizing agent CCCP, comparing the pVV16 (empty vector) control (*black*), Rv1339-expressing (*red*) and Rv1339 D180-expressing (*gray*) *M. smegmatis* mc^2^155 strains. *D*, spot dilution assay of the strains grown for 3 days in 7H10 and 7H10 containing 5 μg/ml of malachite green or 0.01% SDS. *E*, volcano plot for the untargeted metabolomic analysis displaying the differential abundance of metabolites in the empty control vector and Rv1339-expressing strains, cut-off log2 onefold change *p* < 0.01 (*green lines*). *F*, abundances in the different strains of selected metabolites involved in lysine and/or peptidoglycan biosynthesis. The experiments were performed in biological duplicates and technical quadruplicates. Unpaired two-tailed Student’s *t* tests were used to compare the data. ∗*p* < 0.05, ∗∗*p* < 0.01, ∗∗∗*p* < 0.001 and ∗∗∗∗*p* < 0.0001. The data are presented as the means ± SDs from two biological replicates and three replicates. CCCP, carbonyl cyanide m-chlorophenyl hydrazine; DiOC_2_, 3,3′ diethyloxicarbocianide chloride.

The best-characterized mycobacterial cAMP-binding receptor proteins are *M. tuberculosis* H37Rv Crp (Rv3676) and Cmr (Rv1675c) (53, 54, 55, 56, 57). These transcription factors regulate various processes ranging from virulence (58, 59, 60) to carbon metabolism (17, 20, 61, 62, 63) and dormancy (54, 64). *M. smegmatis* mc^2^155 genome does not have a Cmr equivalent but does encode two Crp genes, Crp1 (MSMEG_0539) and Crp2 (MSMEG_6189) (65). A previous study investigated the effect of Crp1 deletion or Crp2 overexpression on the transcriptome (66). Many of the genes found to be regulated by these two proteins are involved in bioenergetic processes, solute transport, and carbon catabolism (66). Based on this knowledge, we anticipated that decreased cAMP levels caused by the expression of Rv1339 in *M. smegmatis* mc^2^155 would alter the transcriptome and that this effect could underpin the changes in bioenergetics and bacterial physiology. To investigate this hypothesis, RNA-Seq of the Rv1339-expressing and empty vector control *M. smegmatis* mc^2^155 strains was performed. The bacteria were grown to mid-log phase in 7H9 medium with glycerol and glucose as carbon sources. Differentially expressed genes were then analyzed (Fig. 3 and Table S2, *A* and *B*).

In the Rv1339-expressing strain, which exhibited significantly decreased cAMP levels, 389 genes (310 upregulated and 79 downregulated) were differentially expressed (*p* < 0.05, the full gene list is shown in Table S2, *A* and *B*). Most of the 389 genes displayed increased expression (310/389), and 39 genes were upregulated by at least a log2 fold change of 1.5 (*p* < 0.05) (Fig. 3, *A* and *B*). Conversely, only five genes were downregulated by at least a log2 fold change of −1.5 (*p* < 0.05).

Among the genes that have increased expression in the Rv1339-expressing strain were some previously identified genes in the Crp regulons. These genes are involved in sugar transport and catabolism of carbohydrates as well as respiratory energy metabolism (Table S2*B*). Importantly, we observed an increase in the expression level of genes involved in the ETC (67). Namely, the predicted operon encoding subunits G, F, E, D, C, and B of the type I NADH:menaquinone oxidoreductase (Nuo) (MSMEG_2057, MSMEG_2058, MSMEG_2059, MSMEG_2060, MSMEG_2061, and MSMEG_2062) with log2 fold changes (*p* < 0.05) of 1.62, 1.26, 1.42, 1.49, 1.01, and 1.77, respectively, as well as in four of the five genes encoding the succinate dehydrogenase 1 (Sdh1) complex (MSMEG_0417, MSMEG_0418, MSMEG_0419, and MSMEG_0420) with a log2 fold changes (*p* < 0.05) of 0.47., 2.60, 3.08, and 3.16, respectively. The expression of both nuo and sdh1 gene clusters has been shown to be negatively regulated by Crp1 (66).

MSMEG_4350, encoding dihydrodipicolinate reductase DapB, was also found to be upregulated with a log2 fold change of 1.06 (*p* < 0.05). This enzyme catalyzes the reduction of 2,3-dihydrodipicolinate to 2,3,4,5-tetrahydrodipicolinate (THDP) using NAD(P)H (68, 69, 70). This reaction is the second step in the biosynthesis of meso-diaminopimelic acid (*m*-DAP) that in turn is the precursor of lysine. Interestingly, *m*-DAP is also a component of mycobacterial PG, forming the L-alanyl-γ-D-isoglutamyl-meso-diaminopimelate-D-alanyl-D-alanine pentapeptide that crosslinks the glycan strands of β(1→4)-linked alternating units of GlcNAc and *N*-acetylmuramic acid (MurNAc) (71, 72, 73). Thus, based on this observation, we hypothesized that a perturbation in the expression of dapB could be a readout of an altered PG biosynthesis and could result in cell wall defects and increased cell envelope permeability.

To test our hypothesis, we first characterized the effect of decreased intracellular cAMP concentrations on the membrane potential by employing the membrane-permeable dye 3,3′ diethyloxicarbocianide chloride (DiOC_2_ (3)). The strong uncoupler carbonyl cyanide m-chlorophenyl hydrazine was used as a positive control of disrupted membrane potential (74, 75, 76). DiOC_2_ (3) is a dye that exhibits green fluorescence when monomeric and undergoes a shift to red emission when aggregated. In cells with intact membrane potential DiOC_2_ (3) is mainly aggregated, so the intensity ratio of red over green correlates with membrane potential. Thus, we measured the membrane potential of the *M. smegmatis* mc^2^155 empty vector control, Rv1339-expressing and Rv1339 D180A-expressing strains. As seen in Figure 3, *A* and *C*, decrease in intracellular cAMP levels in the Rv1339-expressing strain was accompanied by a 15% decrease in the membrane potential compared with the levels found in the empty vector control strain. This decrease was not observed in the Rv1339 D180A-expressing strain, indicating that the decrease in membrane potential was brought about by Rv1339 in a catalytically dependent manner.

The observed decreased membrane potential caused by Rv1339 activity could be the results of cell wall alterations. To further investigate changes in cell wall integrity, we determined the sensitivity of the strains to SDS and malachite green, a lipophilic inhibitor of cell growth used to screen for cell wall perturbations in mycobacteria (20, 77) As seen in Figure 3*D*, the Rv1339-expressing strain was more sensitive to malachite green and to SDS than the Rv1339 D180A-expressing and the empty vector control strains. These results indicate that the compromised cell wall integrity observed in the Rv1339-expressing strain requires the enzyme to be catalytically active and support the observation that this strain displays decreased membrane potential.

To gain a deeper insight into the perturbation of the cell wall, we performed an untargeted metabolomic analysis, which provides a direct readout of the physiological state of the bacteria and allowed us to investigate the changes in cellular concentration of PG building blocks. The bacterial strains were cultured to mid-log phase, and intracellular metabolites were then extracted and analyzed by LC-MS. This analysis showed that the decrease in cAMP levels in the Rv1339-expressing strain was associated with substantial alterations in the level of compounds involved in PG and lysine biosynthesis and catabolism (Fig. 3, *E* and *F* and Table S4). These compounds were confirmed by MS/MS to be GlcNAc-1-phosphate (GlcNAc-1-P; fourfold increase in the Rv1339-expressing strain), alanyl-alanine (1.5-fold increase), THDP (4-fold increase), diaminopimelic acid (2.2-fold decrease), lysine (1.2-fold decrease), and *N*-acetyl lysine (onefold decrease) (Fig. 3, *E* and *F*). These alterations were not observed in the Rv1339 D180A-expressing bacteria (Fig. 3*F*). The accumulation of THDP correlates with the increased expression of dapB observed in the transcriptomics experiment, as THDP is the product of DapB activity. GlcNAc-1-P and D-alanyl-D-alanine are both early PG precursors. In creating the sugar-peptide building blocks of PG, GlcNAc-1-P is uridylylated to form UDP-GlcNAc, which is subsequently transformed into UDP-MurNAc. Then, the pentapeptide is assembled onto UDP-MurNAc in a stepwise manner, first L-Ala, then D-Glu, next m-DAP, and finally D-alanyl-D-alanine. Therefore, the observed accumulation of GlcNAc-1-P and D-alanyl-D-alanine could be explained by a disruption of PG biosynthesis leading to accumulation of precursors. Contrastingly, levels of m-DAP, which is also a component of the PG pentapeptide, were found to be decreased 2.2-fold in the Rv1339-expressing strain. However, besides being involved in PG synthesis, m-DAP is the precursor of lysine, so decrease in its pool size (and that of lysine and *N*-acetyl-lysine) could be due to increased activity of metabolic pathways downstream from lysine. In order to test this hypothesis, we measured metabolite turnover through the incorporation of ^13^C at half-doubling time. We indeed found increased turnover of DAP, lysine, and *N*-acetyl-lysine in the Rv1339-expressing strain (Fig. S3). Additionally, we observed decreased turnover of alanyl-alanine, a fact that would be consistent with accumulation due to defects in PG biosynthesis.

Taken together, these findings show that decreased intracellular cAMP levels result in an altered cAMP regulon and alterations in PG biosynthesis, leading to an increase in cell envelope permeability. We can thus hypothesize that the latter observation should result in changes in drug resistance and/or tolerance to antimicrobials that are known to target cell wall synthesis.

### A decrease in intracellular cAMP levels leads to a decrease in tolerance, but not in resistance, to antibiotics targeting cell wall synthesis

To test the hypothesis that defects in PG biosynthesis caused by the expression of Rv1339 lead to changes in AMR and/or antimicrobial tolerance profiles, we sought to determine the minimal inhibitory concentrations (MIC_50_) and perform time-kill assays of antimicrobials that are known to target cell wall synthesis. We hypothesized that with a compromised PG, antimicrobials that target cell wall assembly should impair the survival of the Rv1339-expressing *M. smegmatis* mc^2^155 strain, relative to the empty vector control and Rv1339 D180A-expressing *M. smegmatis* mc^2^155 strains. We selected three antimicrobials: D-cycloserine (DCS), vancomycin, and ethambutol. DCS blocks bacterial growth by inhibiting the two enzymes responsible for the synthesis of D-alanyl-D-alanine from L-alanine: alanine racemase and D-alanyl:D-alanine ligase (78, 79, 80). Vancomycin binds to the D-alanyl-D-alanine moiety of the PG pentapeptide interfering with PG polymerization (81, 82). Ethambutol inhibits the arabinosyltransferases involved in the synthesis and polymerization of the cell wall components lipoarabinomannan and arabinogalactan (71, 83, 84, 85, 86, 87, 88).

**Figure 4:**
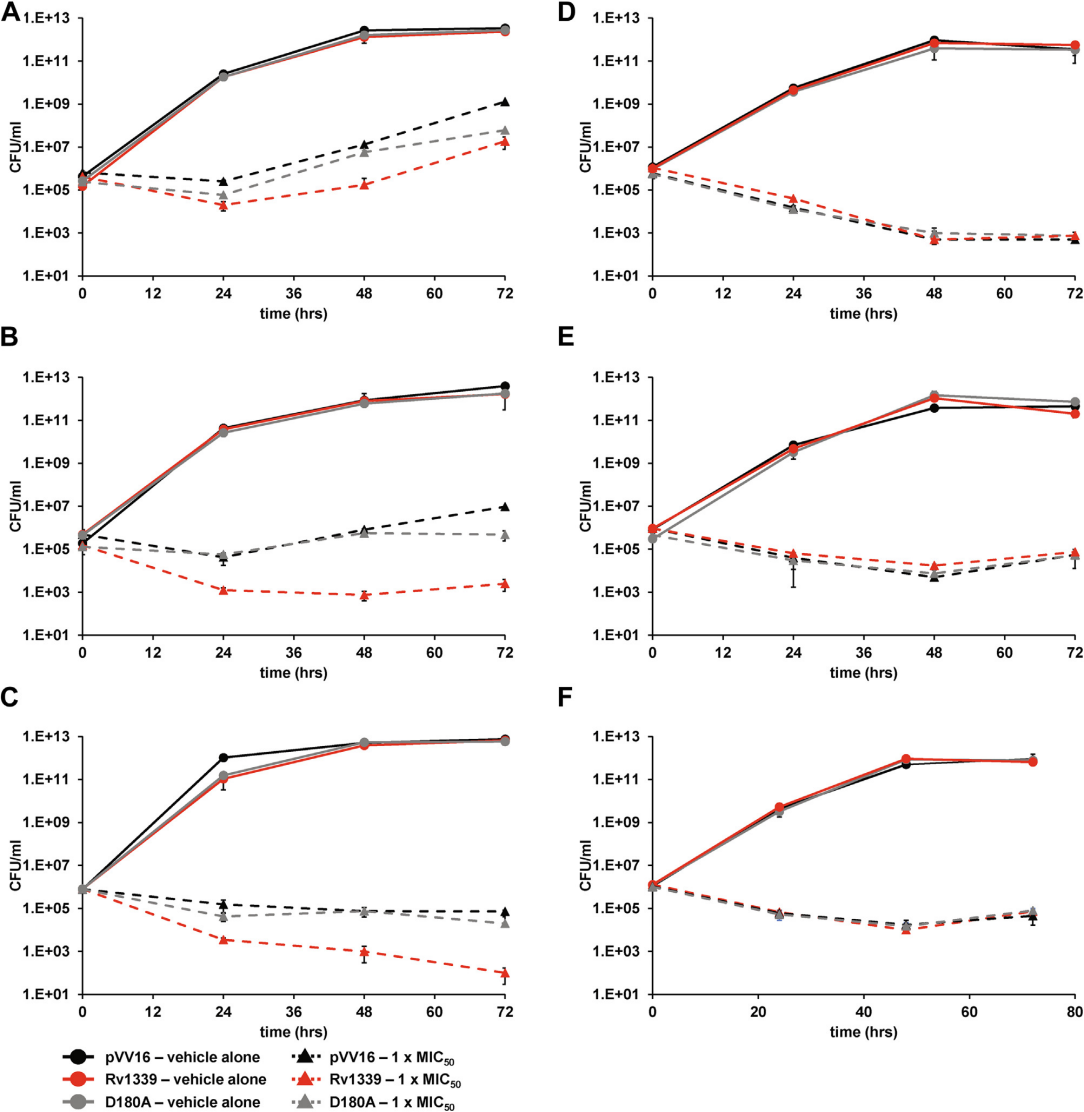
**Expression of Rv1339 reduces tolerance to antibiotics targeting cell wall biosynthesis.** Time-kill assay experiments of the pVV16 (empty vector) control, Rv1339-expressing and Rv1339 D180A-expressing *M. smegmatis* mc^2^155 strains in the presence of three cell wall-targeting antibiotics: D-cycloserine (*A*), vancomycin (*B*), and ethambutol (*C*) compared to control antibiotics with other targets: levofloxacin (*D*), ciprofloxacin (*E*), and rifampin (*F*). *Circles* and *solid lines* indicate vehicle alone and *triangles* and *dashed lines* 1 × MIC_50_. The data are presented as the means ± SDs from two biological replicates and three technical replicates.

We first determined the MIC_50_ of the antimicrobials in the empty vector control, Rv1339-expressing and Rv1339 D180A-expressing *M. smegmatis* mc^2^155 strains. No differences in the MIC_50_ values were observed (Table S3), indicating that changes in intracellular cAMP levels did not alter the resistance profile of the bacteria. We then investigated whether changes in cAMP levels altered antimicrobial tolerance by measuring the viability of the strains in response to treatment with DCS, vancomycin, or ethambutol in a time-kill assay using vehicle alone (0 × MIC_50_) or 1 × MIC_50_ (Fig. 4). In accordance with our previous data (Fig. 2*A*), there was no major growth rate difference between the strains at 0× MIC_50_. However, at 1 × MIC_50_, we observed a decrease in the viable cell count of the Rv1339-expressing strain relative to the empty vector control strain. Effectively, in the presence of DCS, after 48 h of exposure to the 1 × MIC_50_ treatment, a 2-log10 colony-forming units (CFU)/ml reduction in viable cells was observed in Rv1339-expressing strain relative to the empty vector control strain (Fig. 4*A*). This difference was less pronounced after 72 h exposure to 1 × MIC_50_ and could be explained by the emergence of resistant bacteria or antibiotic degradation. A more pronounced effect was observed in the presence of either vancomycin or ethambutol, with a 3-log10 CFU/ml reduction in the viable cell count after 72 h of exposure to these antimicrobials at 1 × MIC_50_ (Fig. 4, *B* and *C*).

In order to determine if those changes are restricted to cell wall targeting antimicrobials, we determined MIC_50_ and performed time-kill experiments of antimicrobials that target other cellular processes. We selected rifampin, levofloxacin, and ciprofloxacin. Rifampin inhibits the elongation of mRNA by binding to the β-subunit of the RNA polymerase (89). Levofloxacin and ciprofloxacin inhibit bacterial DNA synthesis by binding to topoisomerase IV and DNA gyrase (90, 91). Decreased cAMP levels had no effect on the MIC_50_ or on antimicrobial tolerance in these strains (Table S3 and Fig. 4, *D*–*F*). These observations support our hypothesis that the decreased antimicrobial tolerance we observe as a result of reduced cAMP levels is specific to cell wall targeting antimicrobials.

Although the involvement of cAMP in antimicrobial susceptibility in pathogenic gram-negative bacteria has been previously elucidated (9, 92), our data provide the first description of the consequences of reducing the cAMP pool in mycobacteria. This manipulation was achieved without the need of creating gene KOs, simply *via* the constitutive plasmid expression of Rv1339, a previously uncharacterized cyclic nucleotide PDE. We have shown that depleting cAMP compromises cell wall integrity and membrane potential. It is very probable that depletion of cAMP affects PG assembly in other ways than the ones we have characterized here. For example, membrane potential is crucial for the activity of MurJ, the flippase that exports the PG precursor lipid II to the periplasm (93).

In line with our data, a recent study showed that the inactivation of *rv1339* or expression of a mutant *rv1339* allele increased stress resistance in *Mycobacterium canettii* (94). Also, *M. tuberculosis* strains containing point mutations or transposon insertions in *rv1339* exhibit increased resistance to antitubercular compounds (95, 96). These observations suggest that loss of Rv1339 activity renders bacterial less permeable. Moreover, single point mutations in *rv1339* have been found to confer resistance to the entire class of imidazolidines, which are ATP-depleting compounds that are structurally related to the antitubercular drug Q203(96). A possible explanation is that these mutations abrogate Rv1339 activity leading to a reduction in ATP demand that compensates for the effects of ATP depletion.

Additionally, we provide circumstantial evidence that decreasing cAMP levels increases the efficacy of cell wall targeting antimicrobials. These effects are likely the result of compromised cell wall integrity and perhaps also ETC. Therefore, there is a strong incentive to further investigate the validity of targeting the cAMP signaling system in mycobacteria, which might provide insights into increasing the susceptibility of these bacteria to antimicrobial treatment or reducing the development of resistance. Overall, this study provides crucial new mechanistic evidence that the cAMP signaling pathway can be an effective drug target, and it suggests that it may be most vulnerable to combination therapies that could potentially be employed to eradicate persisters.

## Experimental procedures

### Bacterial strains and culture conditions

*M. smegmatis* mc^2^155, parental *M. bovis* BCG, and *M. bovis* BCG Δ*rv0805* were used in this study. The mycobacteria were cultured to the midexponential phase in 7H9 medium supplemented with 0.5 g/l fraction V (bovine serum albumin), 0.05% tyloxapol, 0.2% dextrose, 0.2% glycerol, and 10 mM NaCl. For the time-to-kill assays and metabolomic profiling studies, the mycobacteria were cultured on 7H10 agar supplemented with 0.5 g/l fraction V (bovine serum albumin), 0.2% dextrose, 0.2% glycerol, and 10 mM NaCl. Throughout the study, the mycobacteria were cultured in static or shaking incubators at 180 rpm and 37 °C. The *M. bovis* BCG strains were kindly provided by Prof. Sandhya Visweswariah from the Indian Institute of Science, Bangalore, India.

### Plasmid constructs

*rv1339* was cloned into pVV16 by isothermal assembly using NEBuilder Assembly Master Mix (NEB).

The open vector and insert were obtained by PCR using KAPA HiFi HotStart (Roche) and the following primers: open_pVV16_f (5′-AAGCTTCACCACCACCACCACCACTGACAG-3′), open_pVV16_r (5′-CAT ATG GAA GTG ATT CCT CCG GAT CGG GGA TG-3′), Rv1339_pVV16_f (5′-CCGATCCGGAGGAATCACTTCCATATGCGTCGATGTA TTCCGCATCGTT-3′), and Rv1339_pVV16_r (5′-GTCAGT GGTGGTGGTGGTGGTGAAGCTTGCCGGCTCGCCGGA CTTCG-3′).

Insert and vector PCR products were purified prior to assembly using the QIAquick PCR Purification Kit (Qiagen). Site-directed mutagenesis to obtain the Rv1339 D180A expression construct was achieved by PCR using the complementary primers Rv1339_D180A_f (5′-CTCGCGGCG TCGCCGTTTTCCTCTGC-3′) and Rv1339_D180A_r (5′-GCAGAGGAAAACGGCGACGCCGCGAG-3′) followed by template DNA restriction digestion with DpnI (NEB). The constructs were verified by Sanger sequencing. *M. smegmatis* mc^2^155 bacteria were transformed by electroporation using standard methods. The transformants were plated on 7H10 using hygromycin (50 μg ml^−1^) and kanamycin (25 μg ml^−1^) as the selection markers.

### Preparation of clear soluble lysate

Cultured *M. bovis* BCG cells were aliquoted into 50 ml Falcon tubes under a class II hood and sealed. The cells were then centrifuged at 3000*g*. All centrifugation steps were performed at 4 °C for 5 min. The supernatant was discarded, and the pellet was washed three times with 20 mM Tris–HCl (pH 7.5) and 50 mM NaCl containing a protease inhibitor (Sigma fast). One milliliter aliquots of washed bacteria were transferred to 2 ml microtubes containing 200 μl of 0.1 mm acid-washed soluble lysate, and 200 μl aliquots of the resulting mixture were transferred to Eppendorf tubes. These aliquots were then frozen in liquid nitrogen and maintained at −80 °C for further analysis.

### Chromatography conditions for cAMP PDE activity enrichment

All column purification steps of *M. bovis* BCG lysates were performed using ÄKTA systems at 4 °C. The systems were operated using Unicorn manager software (GE Healthcare). The Capto Q anion-exchange column separates proteins along an NaCl gradient. Low (30 mM) and high (600 mM) concentrations of NaCl with 20 mM Tris–HCl (pH 7.5) were used as buffer A, and size-exclusion chromatography (SEC) was performed using a 24 ml Superdex 200 10/300 column (GE Healthcare) with 20 mM Tris (pH 7.5) and 30 mM NaCl.

### PDE activity assay for the identification of candidate enzymes

Ten microliters (∼1–10 μg) of cell lysate or fractionated lysate was incubated with 2 μl of water, 2 μl of 10× PDE buffer (20 mM MgCl_2_, 200 mM Tris–HCl pH 9.0, and 1000 mM NaCl), and 6 μl of 100 mM (for TLC) or 25 mM (for LC-MS) cyclic AMP or water (as a control) at 37 °C for 16 h. For the 5′-AMP controls, 6 μl of 5′-AMP (10 mM) was added at the same concentrations selected for cAMP. All PDE reactions were performed in Eppendorf tubes.

### TLC to monitor cAMP hydrolysis

Silica gel 60 F254 TLC plates were cut to the required size of less than 15 × 10 cm. The 50 ml mobile phase consisted of 70:30 ethanol/H_2_O 0.2 M ammonium bicarbonate. After migration, the results were revealed by treating the plates with a shearing a solution of 5% phosphomolybdic acid dissolved in ethanol and heating them at 150 °C for 5 to 10 min.

### Identification of the PDE candidate by proteomics analysis

The fraction displaying PDE activity, as determined by TLC after Capto Q and SEC column purification, and the fractions before and after this fraction were run on an SDS gel as described previously and stained with InstantBlue Coomassie stain. The gel lanes were cut out, reduced, alkylated, and digested with trypsin using the ProteaseMAX Surfactant protocol. The samples were then loaded and run on a SYNAPT Q-ToF mass spectrometer. The observed proteins were correlated with the protein IDs and potential annotations in the UniProt database.

### Phylogenetic analysis

Amino acid sequences were aligned with MUSCLE (97). The best-fit amino acid substitution model for the alignment was LG+R6, identified by ModelFinder (98). The trees were calculated using IQ-Tree (1.6.11 (99)) with 100 bootstrap replicates and visualized using Dendroscope (https://github.com/husonlab/dendroscope3) (100). The selected solved 3D structures are indicated by diamonds and labeled with their Protein Data Bank accession codes. The characterized members of the families were as follows (their UniProt and Protein Data Bank accession codes are shown in brackets): *Aliivibrio fischeri* cpdP (Q56686), *Yersinia pestis* cpdP (Q8ZD92), *Saccharomyces cerevisiae* PDE1 (P22434; 4OJV), *E. coli* phosphotriesterase homology protein (PhP; P45548; 1BF6), *Homo sapiens* zinc PDE ELAC protein 1 (Q9H777; 3ZWF), *E. coli* ribonuclease BN (Rbn; P0A8V0; 2CBN), *M. tuberculosis* ribonuclease Z (P9WGZ5), *B. subtilis* ribonuclease Z (P54548; 1Y44), *B. subtilis* metallohydrolase YhfI (O07607; 6KNS), *Streptomyces coelicolor* (SCO2908), *C. glutamicum* CpdA a.k.a. Cgl2508 (Q8NMQ7), and *M. tuberculosis* H37Rv Rv1339 (P9WGC1).

### Bacterial growth assay

Thirty milliliter square bottles were prepared by adding 10 ml of 7H9 medium and incubating the bottles in a static incubator for 12 h to allow the media to reach a temperature of 37 °C. A preculture was prepared by adding 100 μl of bacteria stored at −80 °C to 10 ml of culture medium. Once the preculture reached the mid-log phase, the growth curve was initiated. Specifically, 125 ml conical flasks containing 25 ml of 7H9 medium were inoculated with mycobacteria (from the preculture) at an *A*_600_ of 0.001 and then incubated at 37 °C in a shaking incubator set to 180 rpm. The *A*_600_ was monitored over 80 h.

### His-tagged protein analysis of mycobacteria

To confirm the expression of Rv1339 or Rv1339 D180A, *M. smegmatis* mc^2^155 strains were grown in 7H9 medium as previously described. The bacterial cultures were centrifuged at 3000*g* and 4 °C for 10 min to harvest the bacteria. The pellets were washed three times with 20 mM Tris–HCl and 50 mM NaCl (pH 7.5) and then centrifuged at 3000*g* and 4 °C for 10 min. The pellets were suspended in 1.5 ml of lysis buffer containing a protease inhibitor cocktail (Sigma–Aldrich) and transferred to an O-ring tube containing 100 μl of 0.1 mm acid-washed zirconia beads. All the samples were subjected to bead beating two times with an MP FastPrep-24 homogenizer (MP pharmaceuticals) for 30 s at 0.6 m/second. The samples were then centrifuged at 11,000*g* for 10 min. The supernatant was transferred to an Eppendorf tube, and the protein concentration was determined using a Nanodrop Lite Spectrophotometer (Thermo Fisher Scientific) and bicinchoninic acid (BCA) assay. All the samples were diluted with lysis buffer to ensure equal protein concentrations, added to 5× SDS loading buffer (0.25 M Tris–HCl pH 6.8, 15% SDS, 50% glycerol, 25% β-mercaptoethanol, and 0.01% bromophenol blue), and boiled at 100 °C for 5 min. Subsequently, 50 μg of each sample was loaded onto an SDS-PAGE gel, and the gel was then run at 150 V and 40 mA for 1 h in a tank filled with SDS running buffer (3% Tris, 14.4% glycine, and 1% SDS).

After SDS-PAGE was performed, the gel was transferred to a nitrocellulose membrane (Novex) at 20 V and 400 mA for 1 h at room temperature (RT). The tank was filled with transfer buffer containing 10× SDS (3% Tris, 14.4% glycine, and 1% SDS), methanol, and ddH_2_O at a ratio of 1:2:7. The nonspecific sites on the nitrocellulose membrane were blocked overnight with 10 ml of 5% skimmed milk (Marvell) suspended in buffer composed of 0.06% Tris base, 0.88% NaCl, and 0.1% Tween-20 (pH 7.5). The membrane was washed with buffer, incubated for 1 h with a mouse horeseradish peroxidase α-His tag antibody (1:1000 in 5% milk) (BioLegend), and developed. The Rv1339 protein was visualized with a FujiFilm LAS-3000 Image Reader and the Amersham ECL Western blotting analysis system (GE Healthcare).

### *In vitro* PDE activity of cell-free extracts

*M. smegmatis* mc^2^155 strains were grown in 7H9 medium at 37 °C as described previously. The pellets were washed three times with 20 mM Tris–HCl and 50 mM NaCl (pH 7.5) and then centrifuged at 3000*g* and 4 °C for 10 min. The pellets were suspended in 1.5 ml of lysis buffer containing a protease inhibitor cocktail (Sigma–Aldrich) and transferred into an O-ring tube containing 100 μl of 0.1 mm acid-washed zirconia beads. All the samples were subjected to bead beating three times with an MP FastPrep-24 homogenizer (MP pharmaceuticals) set at 6.0 m/second for 60 s. The samples were then centrifuged at 11,000*g* for 10 min. The supernatant was transferred to an Eppendorf tube, and the protein concentration was determined using a BCA assay kit (Thermo) (101, 102). Stock solutions of nucleotides were prepared in ddH_2_O at a concentration of 10 mM. For the assay conducted in a total reaction volume of 50 μl, 30 μg of cell-free extract was incubated with 10 μM 2′,3′-cAMP; 3′,5′-cAMP; 3′,5′-cGMP; 2′,3′-cCMP; ATP; ADP; AMP; UMP; c-di-AMP; or c-di-GMP in 20 mM Tris–HCl pH 7.5, 10 mM MgCl_2_, and 100 mM NaCl. At time 0 and after 30 min of incubation at 37 °C, the reaction was quenched with 50 μl of quenching solution composed of acetonitrile/methanol/H_2_O (2:2:1) precooled to 4 °C on ice, 100 μl of acetonitrile was then added and the product of the reaction was spun for 10 min at 17,000*g* at 4 °C. The supernatants, which contained the products of the reactions, were injected into the LC-MS/MS system. The relative activity at time 0 was calculated by dividing the abundance of the substrates at the time point of 30 min by the abundance of the substrates at time 0. The experiments were performed in biological triplicates and technical duplicates. Unpaired two-tailed Student's *t* tests were used to compare values, with *p* < 0.05 considered significant. The SDs were calculated according to the error propagation in the calculated ratios (103).

### Determination of the MIC_50_ by a resazurin microtiter assay

The MIC_50_ was determined using the resazurin microtiter assay according to the NCCLS guidelines (104) and Palomino *et al.* (2002) (105). Antibiotic stock solutions of the tested compounds were prepared to yield the target concentrations for testing. Microdilution assays were performed in 96-well plates. Twofold serial dilutions were performed to obtain the final drug concentration, which ranged from 800 to 150 μg/ml (*D*-cycloserine), 10 to 0.04 μg/ml (ethambutol), and 100 to 0.4 μg/ml (vancomycin), from 400 to 1.56 μg/ml (rifampin) and from 4 to 0.016 μg/ml (levofloxacin and ciprofloxacin). To obtain the bacterial inoculum, the pVV16 (empty vector) control, Rv1339-expressing and Rv1339 D180A-expressing *M. smegmatis* strains were grown to the mid-log phase (*A*_600_ ∼ 0.5) and diluted 1:1000 with 7H9 medium. Fifty microliters of the standardized bacterial inoculum and 50 μl of 7H9 medium were added to each well of a 96-well plate. The plates were then incubated for 48 h at 37 °C with 5% CO_2_. Subsequently, 30 μl of 0.01% resazurin was added, and the mixture was maintained at 37 °C with 5% CO_2_. After color development for 24 h, the wells were read. The MIC_50_ was defined as the lowest concentration that inhibited the growth of 50% of bacteria.

### Time-kill assays

Strains were grown to the mid-log phase (*A*_600_ ∼ 0.5) and diluted 1:100 to yield concentrations of ∼10^5^-10^6^ CFUs/ml in Middlebrook 7H9 medium. Antibiotics were added to each sample at defined concentrations, and the bacterial samples were collected before the addition of the antibiotic and at 24, 48, and 72 h after antibiotic challenge. To determine the number of viable cells, CFUs were determined through serial 10-fold dilutions using 20 μl of culture and 180 μl of 7H9 medium. Twenty microliters of each dilution were plated on 7H10 agar (Sigma–Aldrich). All the plates were incubated at 37 °C for 4 days before the colonies were counted.

### ATP/ADP/AMP ratios and adenylate energy charge in *M. smegmatis* lysates

Intracellular ATP, ADP, and AMP were extracted as described earlier (106). Briefly, strains were grown in 7H9 medium at 37 °C as described previously. The pellets were suspended in 0.5 ml of fresh culture medium twice, pooled, and transferred into a new Eppendorf tube. The suspensions were then centrifuged for 10 min at 1500*g* at 4 °C, and the supernatants were discarded. To the pellets was added an extraction solution composed of acetonitrile/methanol/H_2_O (2:2:1) precooled to 4 °C. After mixing up and down, the suspended cells were transferred into an O-ring tube containing 100 μl of 0.1 mm acid-washed zirconia beads. All the samples were subjected to bead beating two times with an MP FastPrep-24 homogenizer (MP pharmaceuticals) set at 6.0 m/second for 60 s, including 5 min of resting on ice between rounds of lysis. The lysates were then heated at 98 °C for 10 min to inactivate hydrolase activity. The whole lysates were then allowed to rest on ice for 10 min and filtered once through 0.22 μm Spin-X column filters (CoStar). The column flow-through was then transferred to LC-MS V-shaped vials (Agilent; 5188-2788), and a 3 μl aliquot was injected into the LC-MS instrument.

The AEC was calculated according to the formula determined by Atkinson (47, 48)

$$\text{AEC}=\frac{\left(ATP+\frac{1}{2}ADP\right)}{\left(AMP+ADP+ATP\right)}$$

The experiments were performed in biological duplicates and technical triplicates. Unpaired two-tailed Student's *t* tests were used to compare values, with *p* < 0.05 considered significant.

### NADH/NAD^+^ ratio in *M. smegmatis* strains

Reaction buffer containing 0.2 M bicine (pH = 8.0, Sigma), 20% ethanol, 8 mM EDTA, 6.6 mM phenazine ethosulfate (Sigma), and 0.84 mM thiazolyl blue tetrazolium bromide (MTT, Sigma) was prepared away from light. NAD (NAD^+^ and NADH, both from Sigma) standards were prepared in ddH_2_O with different concentration ranges (NAD^+^: 0–2.5 μM; NADH: 0–0.1 μM). Alcohol dehydrogenase (from *S. cerevisiae*, purchased from Sigma) solution was prepared at 1 mg/ml (314 units/ml) in ddH_2_O. *M. smegmatis* strains were harvested from 1 ml culture aliquots in microtubes by centrifugation at 15,000*g* for 10 min. Pellets were cooled on ice and resuspended in 250 μl of either 0.2 M HCl (NAD^+^ samples) or 0.2 M NaOH (NADH samples) and heated to 55 °C for 10 min. Samples were neutralized with 0.2 M NaOH (NAD^+^ samples) or HCl (NADH samples), and the pH was verified by using pH strips to be close to pH = 7. The neutralized samples were centrifuged at 12,000 rpm and 4 °C for 10 min. Clear-bottomed black 96-well plates (Greiner Bio-One) were loaded with 80 μl aliquots of standards and samples, and 100 μl of reaction buffer was added to each well. The plates were incubated in the dark for 5 min, after which 20 μl of enzyme solution was loaded into each well, and the plates were immediately introduced to a plate reader for kinetic measurement at λ570 nm every 30 s over 10 min. Data were retrieved and processed by using Microsoft Excel. Standard curves with different concentrations of NADH/NAD^+^ were produced to correlate the changes in the absorbance per minute with increasing concentrations and were then used to quantify concentrations of NADH/NAD^+^ in samples.

### Analysis of the membrane potential

*M. smegmatis strains* were grown to an absorbance of ∼0.6, centrifuged, and suspended in 7H9 media. One milliliter aliquots were collected at various time points. The samples were washed with 7H9 media without albumin, suspended in 1 ml of 7H9 media without albumin, and exposed to 15 μM DiOC_2_ with or without 5 μM carbonyl cyanide m-chlorophenyl hydrazine at for 20 min at RT in the dark. The samples were then washed with 7H9 media without albumin and suspended in the same media. The fluorescence was monitored using a Berthold Mithras LB 940 plate reader: green 488/530 nm and red 488/610 nm. The membrane potential was calculated as the ratio of red to green fluorescence.

### Monitoring cell wall permeability

Approximately 10^6^ cells (based on optical density measurements) from mid-log phase cultures of each strain were spotted onto 7H10 agar plates containing 5 μg/ml of malachite green or 0.01% SDS. The plates were incubated at 37 °C for 3 days and photographed.

### RNA extraction and RNA-Seq

*M. smegmatis* mc^2^155 strains were grown in 7H9 medium at 37 °C as described previously. The bacteria were grown to the mid-log phase (light transmittance at 600 nm of 0.6) and washed twice with cold PBS at 4 °C. Total RNA was extracted according to the FastRNA Pro Blue kit manual (MP). For the removal of genomic DNA, the samples were treated once with RNase-free DNase (Promega) for 3 h at 37 °C and purified using RNAeasy columns (Qiagen) according to the manufacturer’s instructions. The RNA quantity and quality were assessed using a NanoDrop 1000 spectrophotometer (Thermo Fisher Scientific, Inc) and an Agilent 2100 Bioanalyzer with an RNA 6000 Nano LabChip kit (Agilent Technologies, Ltd). All the samples displayed a 260/280 ratio greater than 2.0 and RNA integrity numbers greater than 9.1. RNA was sequenced using an Illumina HiSeq4000 sequencer in the Imperial BRC Genomics Facility at Imperial College London. Samples were sequenced to obtain paired-end reads with a minimum of 75 bp in length.

### Complementary DNA library preparation and RNA-seq data

Complementary DNA was generated from RNA *via* rRNA depletion using the NEBNext rRNA Depletion Kit (Bacteria) (NEB #E7850, NEB #E7860). RNA and library quality control and quantification procedures were performed on a Tapestation 4200 (Agilent) according to the recommendations of the manufacturer. The sequence quality was checked using FastQC (v0.11.5, http://www.bioinformatics.babraham.ac.uk/projects/fastqc/). All the sequences passed quality control, reads were trimmed using fastp (v0.22.0 (107)), and aligned to the *M. smegmatis* mc^2^155 reference genome (GCF_000015005.1 assembly, NCBI; using the Burrows-Wheeler Transform (BWA) sequence aligner (v0.7.15 (108, 109)). Samtools (v1.2, (110)) was used for file format conversion and sorting of reads. The read counts were calculated at the gene level, in R (v3.6.0; https://cran.r-project.org/bin/windows/base/old/3.6.0/), using Feature Counts from the Rsubread package (v2.0.1 (111, 112)) and the *M. smegmatis* mc^2^155 annotation (GCF_000015005.1 assembly, NCBI). The sequences were also aligned to the *M. tuberculosis* H37Rv reference genome (GCF_000195955.2_ASM19595v2 assembly, NCBI) and annotated using the *M. tuberculosis* H37Rv annotation (GCF_000195955.2_ASM19595v2 assembly, NCBI) to determine the levels of *rv1339* in all the samples. Genes with low counts in all the samples were removed; counts were normalized and transformed using variance stabilizing transformation from the DESeq2 Bioconductor package (v1.26.0 (113)) package, before sample quality was evaluated through principal component analysis. Differential gene expression analysis was performed according to the “Analyzing RNA-Seq with DESeq2” workflow (113). In this analysis, the Rv1339-expressing strain was compared with the empty control vector-expressing strain. Genes were corrected for multiple testing using the Benjamini-Hochberg method, and significant genes were identified using a false discovery rate cut-off of < 0.05 and an absolute log2 fold change of 1.5.

### Bioenergetic analysis using Seahorse XFP

The 7H9 medium was prepared according to the literature (45, 46, 114). The carbon source supplement (glycerol and glucose) was prepared at a final concentration of 2% in ddH_2_O and filtered to ensure that it was sterile.

A Seahorse XFP analysis was performed using methods similar to those applied previously. All reagents were purchased from Agilent Technologies, and all work was performed in a laminar-flow hood. The day before the assay was to be run, the Seahorse XFP cartridge probes were hydrated by filling the utility plate and all border wells with 200 μl of sterile ddH_2_O per well. This utility plate-cartridge unit was then incubated in an airtight container overnight.

A minimum of 2 h before the assay was run, the ddH_2_O was removed and replaced with XFP calibrant solution, and the cartridge was returned to the 37 °C incubator. Subsequently, 20 μl of each substance to be injected was introduced *via* the relevant injection port, and during this process, care was taken to ensure that none of the sample adhered to the sides of the injection port. In the assays performed in this study, the only substance injected was glucose–glycerol, which was injected at a final concentration of 0.2%. Each well of the bacterial cell plate was separately coated with 90 μl of sterile poly-D-lysine and allowed to air dry overnight in a sealed laminar-flow hood. On the day of the assay, the residual poly-D-lysine was removed, and the wells were washed with 90 μl of ddH2O. The water was then removed, and the plate was allowed to air-dry in the laminar-flow hood with the lid off. The bacteria were diluted to a volume of 1 ml with an *A*_600_ of 0.51 (this quantity of bacteria was determined to be within the reliable working range of the Seahorse XFP instrument after extensive optimization). These samples were then centrifuged for 10 min at 15,000*g* at RT to pellet the bacteria, and the supernatant was discarded. The bacteria were then resuspended in unbuffered 7H9 medium and centrifuged for 7 min at RT. The bacteria were resuspended in 1 ml of new unbuffered 7H9 medium. A total of 90 μl of the well-mixed bacterial solution was deposited in each well of the Seahorse XFP bacteria cell plate, and the plate was then centrifuged at 2000*g* for 10 min. Subsequently, 90 μl of unbuffered 7H9 medium was gently added in a dropwise manner to increase the volume to 180 μl.

At least 2 h after calibrant incubation in the utility plate-cartridge unit, the unit was ready for calibration in the Seahorse XFP instrument. Once calibrated, the utility plate was ejected and could be replaced with the bacterial cell plate. The instrument was returned to 37 °C to begin the assay. Each measurement cycle requires 4 min of mixing and 2 min of sensor measurement. The assay consisted of three measurement cycles in the absence of a carbon source. Subsequently, 20 μl of 2% glucose–glycerol was injected at a final concentration of 0.2%, and 12 additional measurements were performed. The bacterial counts were normalized by the CFUs of the different bacterial strains used to inoculate each well. The data were normalized to 10^5^ CFUs.

### Metabolite extraction experiments

For the targeted and untargeted metabolomics profiling studies, the mycobacteria were initially grown in 7H9 medium containing the carbon sources of interest until the *A*_600_ reached approximately 0.8 to 1. The bacteria were then inoculated onto 0.22 μm nitrocellulose filters under vacuum filtration. The mycobacteria-laden filters were subsequently placed on top of chemically equivalent agar media (described before) and allowed to grow at 37 °C for five doubling times to generate sufficient biomass for targeted metabolomics studies. The filters were then transferred to 7H10 plates supplemented with 0.5 g/l fraction V (bovine serum albumin), 0.2% dextrose, 0.2% glycerol, and 10 mM NaCl. The bacteria were metabolically quenched by plunging the filters into the extraction solution composed of acetonitrile/methanol/H_2_O (2:2:1) precooled to 4 °C. Small molecules were extracted by the mechanical lysis of the entire bacteria-containing solution with 0.1 mm acid-washed zirconia beads for 1 min using a FastPrep system (MP Bio) set to 6.0 m/second. The lysates were filtered twice through 0.22 μm Spin-X column filters (CoStar). The bacterial biomass of the individual samples was determined by measuring the residual protein content of the metabolite extracts using the BCA assay kit (Thermo) (101, 102). A 100 μl aliquot of the metabolite solution was then mixed with 100 μl of acetonitrile with 0.2% acetic acid at −20 °C, and the mixture was centrifuged for 10 min at 17,000*g* and 4 °C. The final concentration of 70% acetonitrile was compatible with the starting conditions of HILIC-Z and Diamond Hydride chromatography. The supernatant was then transferred into LC-MS V-shaped vials (Agilent 5188-2788), and a 4 μl aliquot was injected into the LC-MS instrument.

### LC-MS for targeted metabolomic analysis

Aqueous normal-phase liquid chromatography was performed using an Agilent 1290 Infinity II LC system equipped with a binary pump, temperature-controlled autosampler (set at 4 °C), and temperature-controlled column compartment (set at 25 °C) containing a Cogent Diamond Hydride Type C silica column (150 mm × 2.1 mm; dead volume of 315 μl). A flow rate of 0.4 ml/min was used. The elution of polar metabolites was performed using solvent A, which consisted of deionized water (resistivity ∼18 MΩ cm) and 0.2% acetic acid, and solvent B, which consisted of 0.2% acetic acid in acetonitrile. The following gradient was applied at a flow rate of 0.4 ml/min: 0 min, 85% B; 0 to 2 min, 85% B; 3 to 5 min, 80% B; 6 to 7 min, 75% B; 8 to 9 min, 70% B; 10 to 11 min, 50% B; 11.1 to 14 min, 20% B; 14.1 to 25 min, 20% B; and 5-min of re-equilibration at 85% B. Accurate MS was performed using an Agilent Accurate Mass 6545 QTOF apparatus. Dynamic mass axis calibration was achieved by continuous infusion after the chromatography of a reference mass solution using an isocratic pump connected to an electrospray ionization source operated in positive-ion mode. The nozzle and fragmentor voltages were set to 2000 V and 100 V, respectively. The nebulizer pressure was set to 50 psig, and the nitrogen drying gas flow rate was set to 5 l/minute. The drying gas temperature was maintained at 300 °C. The MS acquisition rate was 1.5 spectra/sec, and *m*/*z* data ranging from 50 to 1200 were stored. This instrument enabled accurate mass spectral measurements with an error of less than 5 ppm, a mass resolution ranging from 10,000 to 45,000 over the *m*/*z* range of 121 to 955 atomic mass units, and a 100,000-fold dynamic range with picomolar sensitivity. The data were collected in centroid 4 GHz (extended dynamic range) mode. The detected *m*/*z* data were deemed to represent metabolites, which were identified based on unique accurate mass-retention times and MS/MS fragmentation identifiers for masses exhibiting the expected distribution of accompanying isotopomers. The typical variation in the abundance of most of the metabolites remained between 5% and 10% under these experimental conditions.

### LC-MS for untargeted metabolomic analysis and nucleotide quantification

The data were acquired with an Agilent 1290 Infinity II UHPLC coupled to a 6545 LC/Q-TOF system. Chromatographic separation was performed with an Agilent InfinityLab Poroshell 120 HILIC-Z (2.1 × 100 mm, 2.7 μm (p/n 675775-924)) column. The HILIC-Z methodology was optimized for polar acidic metabolites. For easy and consistent mobile-phase preparation, a concentrated 10× solution consisting of 100 mM ammonium acetate (pH 9.0) in water was prepared to produce mobile phases A and B. Mobile phase A consisted of 10 mM ammonium acetate in water (pH 9) with a 5 μM Agilent InfinityLab deactivator additive (p/n 5191-4506), and mobile phase B consisted of 1.0 mM ammonium acetate (pH 9) in 10:90 (v:v) water/acetonitrile with a 5 μM Agilent InfinityLab deactivator additive (p/n 5191-4506). The following gradient was applied at a flow rate of 0.5 ml/min: 0 min, 100% B; 0 to 11.5 min, 70% B; 11.5 to 15 min, 100% B; 12 to 15 min, 100% B; and 5-min of re-equilibration at 100% B. Accurate MS was performed using an Agilent Accurate Mass 6545 QTOF apparatus. Dynamic mass axis calibration was achieved by continuous infusion after the chromatography of a reference mass solution using an isocratic pump connected to an electrospray ionization source operated in negative-ion mode. The following parameters were used: sheath gas temperature, 300 °C; nebulizer pressure, 40 psig; sheath gas flow, 12 l min^−1^; capillary voltage, 3000 V; nozzle voltage, 0 V; and fragmentor voltage, 115 V. The data were collected in centroid 4 GHz (extended dynamic range) mode.

### Nucleotide standard curve

A stock solution of nucleotides at a concentration of 100 mM in double-distilled water was prepared and serially diluted in a solution of acetonitrile/methanol/H_2_O (2:2:1) to obtain concentrations in the range of 100 to 0.01 μM in technical quadruplicates. A standard curve was established using Agilent Quantitative Analysis B.07.00.

### Stable isotope labeling

Under the experimental conditions described previously using [U-^13^C_3_]-glycerol (99%) and [U-^13^C_6_]-glucose (99%), the extent of ^13^C labeling of each metabolite was determined by dividing the summed peak height ion intensities of all ^13^C-labeled species by the ion intensity of both labeled and unlabeled species using Agilent Profinder version B.8.0.00 service pack 3.

### Statistical analysis

The data are presented as the means ± SDs from at least two biological replicates and three technical replicates per condition. Unpaired two-tailed Student’s *t* tests were used to compare the data and *p* < 0.05 was considered significant.

### Biological safety considerations

The bacteria were handled within a Class-II safety level cabinet equipped with a UV light source and high efficiency particulate air filters for safety considerations and handling of the bacteria aseptically.

## Data availability

The RNA-Seq data are available in GEO under the accession number GSE157084, and the accompanying analysis code is available at https://github.com/ash-omics/cAMP_RNAseq and https://github.com/Nerdobyte/cAMP_RNAseq. All data presented in this study are contained within the article and available from authors upon request.

## Supporting information

This article contains supporting information.

## Conflict of interest

The authors declare that they have no conflicts of interest with the contents of this article.

## Supporting information

Supporting Information

Supplementary Table 2A

Supplementary Table 2B
