# Supplementary material for: Expression of a novel mycobacterial phosphodiesterase successfully lowers cAMP levels resulting in reduced tolerance to cell wall–targeting antimicrobials
Source: J Biol Chem. 2022 Jun 17;298(8):102151. doi: 10.1016/j.jbc.2022.102151 (PMC9293780; doi:10.1016/j.jbc.2022.102151)
Supplement: Supporting Information [file mmc1.docx]

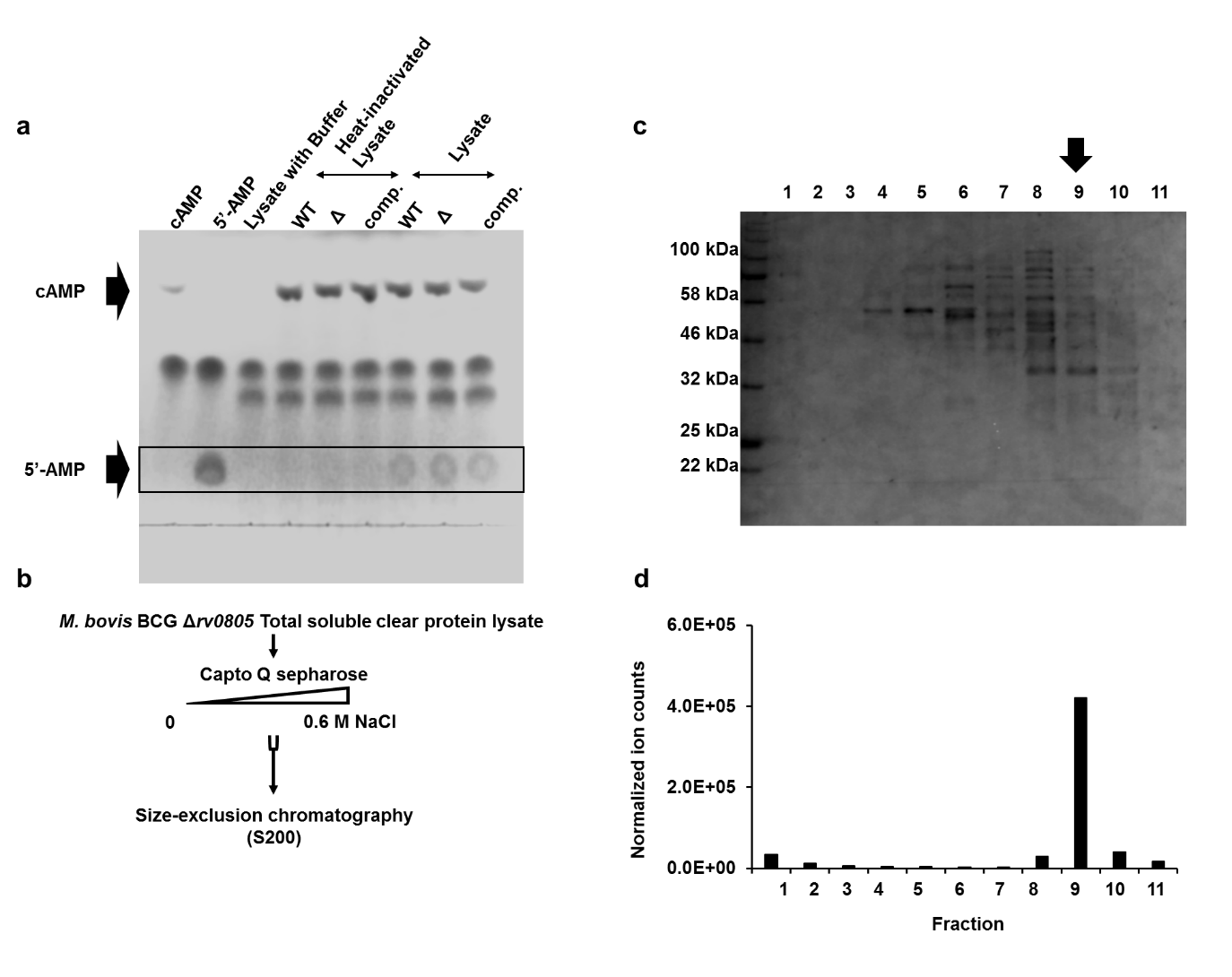


**Supplementary Figure 1: Identification of Rv1339**. **a**. TLC showing the *in vitro* cAMP activity of clear lysate from parental *M. bovis* BCG and strains with deletion (Δ) and complementation (comp.) of the *Rv0805* gene. **b**. Strategy for identifying the PDE activity of the Δ*rv0805* clear lysate using a series of liquid chromatography techniques. **c.** SDS-PAGE showing the protein content of the fractions collected from the last chromatography step. Black arrow indicates the most active fraction towards cAMP hydrolysis. **d**. Bar graph displaying the ion counts normalized to the amount of 5’-AMP protein from the fraction arising from the S200 size-exclusion chromatography.


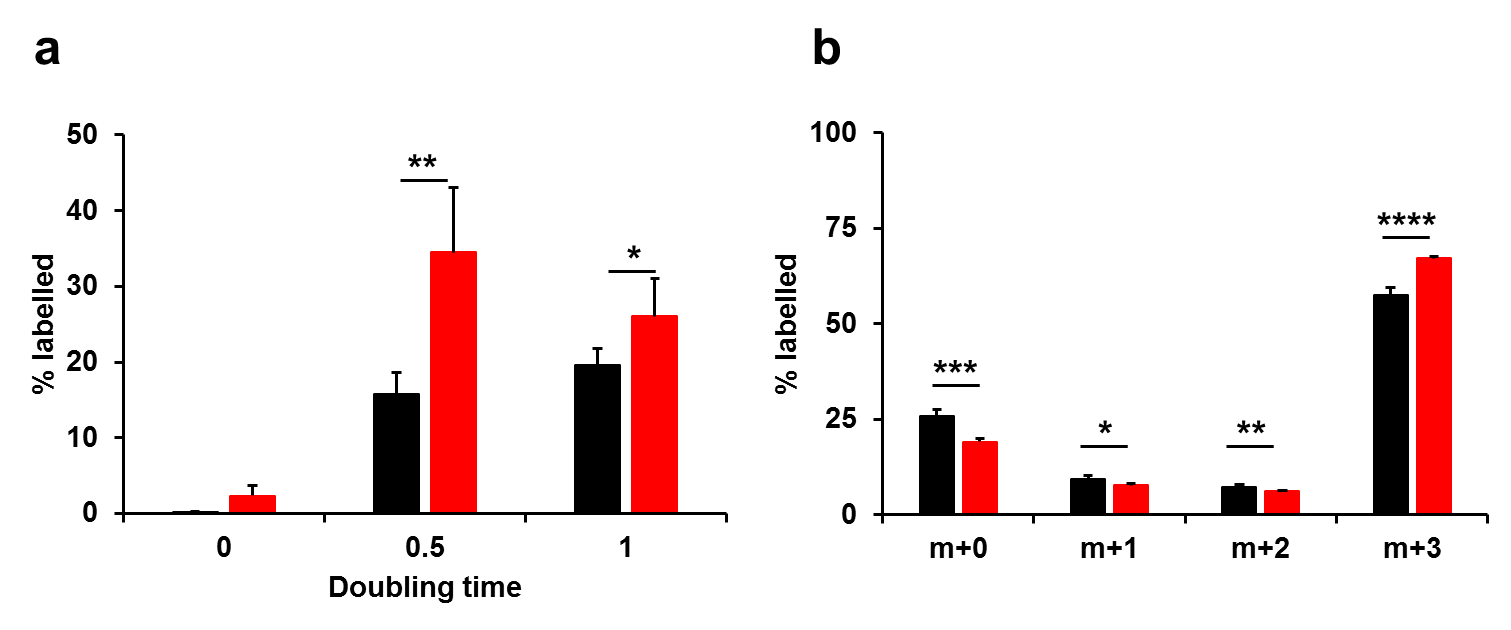


**Supplementary Figure 2: The expression of Rv1339 leads to changes in carbon turnover**. **a.** Percentage of labelled cAMP. **b.** Isotopologue distribution of serine. The data are presented as the means±SDs from two biological replicates and three replicates. Black bars correspond to the *M. smegmatis* mc^2^155 pVV16 strain and the red bars correspond to the *M. smegmatis* mc^2^155 pVV16::r*v1339* strain.


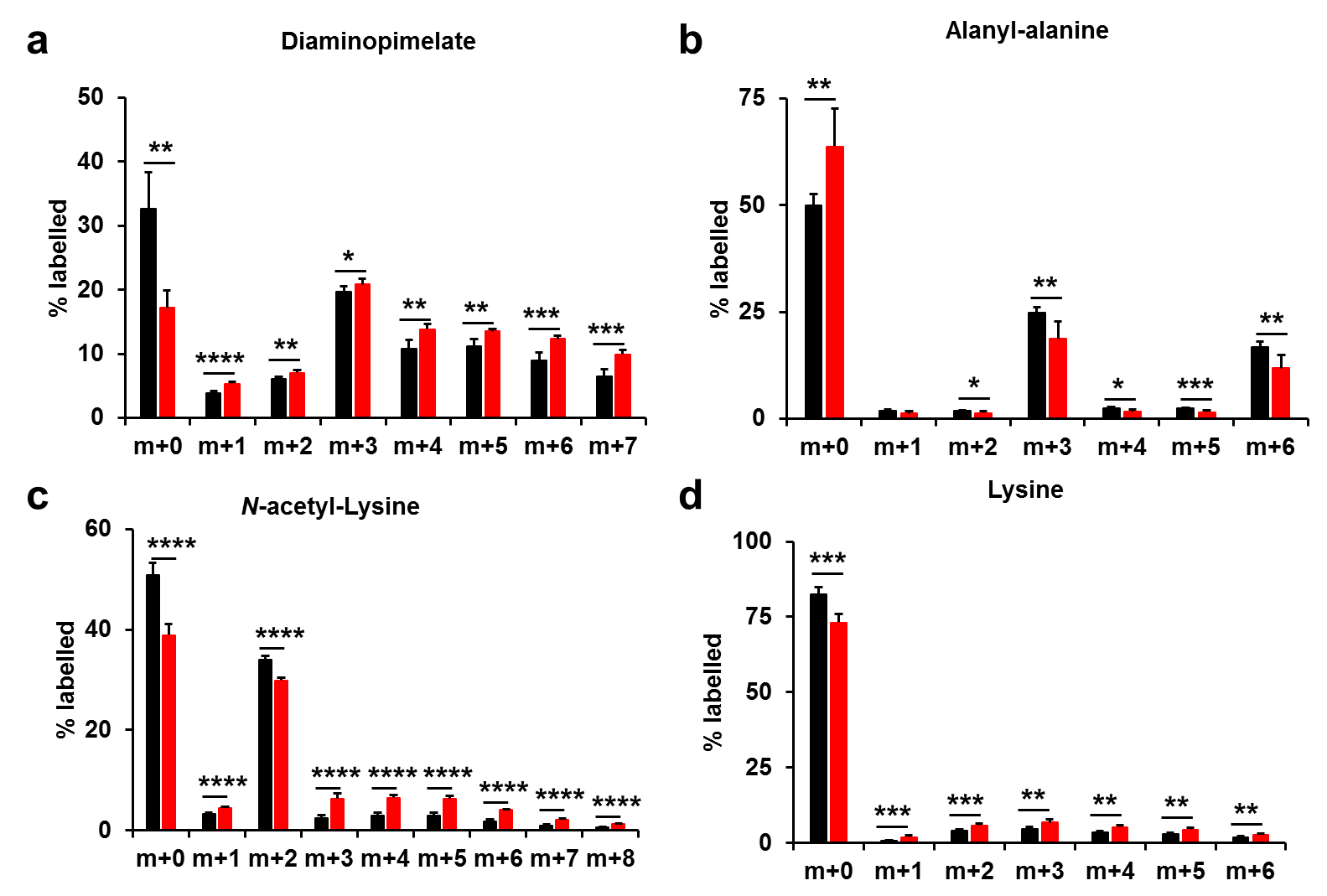


**Supplementary Figure 3:** **The expression of Rv1339 leads to an increase in the turnover of metabolites involved in peptidoglycan synthesis. a**. Isotopologue distribution of meso-2,6-diaminoheptanedioate. **b**. Isotopologue distribution of D-alanyl-D-alanine. **c**. Isotopologue distribution of N-acetyl-lysine. **d.** Isotopologue distribution of lysine. The data are presented as the means±SDs from two biological replicates and three replicates. Black bars correspond to the *M. smegmatis* mc^2^155 pVV16 strain and the red bars correspond to the *M. smegmatis* mc^2^155 pVV16::r*v1339* strain.

**Supplementary Table 1: Doubling time of the *M. smegmatis* mc^2^155 strains used in the study.** The data are presented as the means±SDs from three biological replicates and three technical replicates.

| **Strains** | **Doubling time**  **(hours)** | ***p* value compared**  **with the empty vector-expressing strain** |
| --- | --- | --- |
| *M. smegmatis* pVV16 | 3.03 ± 0.07 | - |
| *M. smegmatis* pVV16::*rv1339* | 3.70 ± 0.21 | 0.001 |
| *M. smegmatis* pVV16::rv1339 D180A | 3.07 ± 0.09 | ns |

**Supplementary Table 2a: List genes in Rv1339-expressing *M. smegmatis* mc^2^155.** RNA sequencing of empty vector control - and Rv1339-expressing *M. smegmatis* mc^2^155 strains in the mid-log phase of growth. The data are presented three biological replicates.

**Supplementary Table 2b: List genes significantly expressed in Rv1339-expressing *M. smegmatis* mc^2^155 with an absolute log2 fold change greater than 1.5.** RNA sequencing of empty vector control - and Rv1339-expressing *M. smegmatis* mc^2^155 strains in the mid-log phase of growth. The data are presented from three biological replicates.

**Supplementary Table 3: MIC_50_ values of the *M. smegmatis* mc^2^155 strains used in the study.** The data are presented as the means±SDs from three biological replicates and three technical replicates.

| **Antibiotics** | **MIC (μg/mL)**  ***M. smegmatis* pVV16** | **MIC (μg/mL)**  ***M. smegmatis* pVV16::*rv1339*** | **MIC (μg/mL)**  ***M. smegmatis* pVV16::*rv1339* D180A** |
| --- | --- | --- | --- |
| D-cycloserine | 400.0 ± 0.0 | 400.0 ± 0.0 | 400.0 ± 0.0 |
| Ethambutol | 1.0 ± 0.0 | 1.0 ± 0.0 | 1.0 ± 0.0 |
| Vancomycin | 12.5 ± 0.0 | 12.5 ± 0.0 | 12.5 ± 0.0 |
| Rifampicin | 50.0 ± 0.0 | 50.0 ± 0.0 | 50.0 ± 0.0 |
| Levofloxacin | 0.25 ± 0.0 | 0.25 ± 0.0 | 0.25 ± 0.0 |
| Ciprofloxacin | 0.25 ± 0.0 | 0.25 ± 0.0 | 0.25 ± 0.0 |

**Supplementary Table 4: List of metabolites measured by LC-MS in the negative and positive ion mode this study.**

| **Compound Name** | **Chemical Formula** | **Mass** | **Retention Time (min)** | **Theoritical *m/z* (neg)** | **Experimental *m/z* (neg)** | **Δppm** |
| --- | --- | --- | --- | --- | --- | --- |
| Serine | C3H7NO3 | 105.042593 | 8.5 | 104.0353 | 104.03536 | -0.6 |
| Lysine | C6H14N2O2 | 146.1055 | 10 | 145.0983 | 145.09813 | 1.2 |
| Alanyl-alanine | C6H12N2O3 | 160.0848 | 5.6 | 159.0775 | 159.0774 | 0.6 |
| *L*-2-aminoadipic acid | C6H11NO4 | 161.0688 | 7.9 | 160.0615 | 160.0614 | 0.6 |
| *L*-2,3,4,5-Tetrahydrodipicolinate | C7H9NO4 | 171.0532 | 5 | 170.0459 | 170.04425 | 9.7 |
| Diaminopimelate | C7H14N2O4 | 190.095357 | 9.2 | 189.0880 | 189.08749 | 3.1 |
| *N*-Acetyl-alpha-*D*-glucosamine 1-phosphate | C8H16NO9P | 301.0563 | 8.4 | 300.049 | 300.04911 | -0.4 |
| ATP | C10H16N5O13P3 | 506.9957 | 11.2 | 505.9885 | 505.98874 | 0.4 |
| ADP | C10H15N5O10P2 | 427.0294 | 9.4 | 426.0221 | 426.02251 | 0.9 |
| AMP | C10H14N5O7P | 347.0631 | 7.2 | 346.0558 | 346.05581 | 0.02 |
| c-di-AMP | C20H24N10O12P2 | 658.105 | 5.6 | 657.0978 | 657.09551 | -3.5 |
| c-di-GMP | C20H24N10O14P2 | 690.0949 | 8.1 | 689.0876 | 689.08777 | 0.2 |
|  |  |  |  | **theoretical *m*/*z* (pos)** | **experimental *m*/*z* (pos)** | **Δppm** |
| 3’,5’-cAMP | C10H12N5O6P | 329.0525 | 3 | 330.0598 | 330.06002 | 0.7 |
| 2’,3’-cAMP | C10H12N5O6P | 329.0525 | 2.5 | 330.0598 | 330.06051 | 2.1 |
| 2’,3’-cCMP | C9H12N3O7P | 305.0413 | 4.8 | 306.0486 | 306.04915 | 1.8 |
| 3’,5’-cGMP | C10H12N5O7P | 345.0474 | 2.2 | 346.0547 | 346.05546 | 2.2 |
| UMP | C9H13N2O9P | 324.0359 | 3.1 | 325.0431 | 325.04341 | 0.9 |

**Supplementary Table 5: Percentage of ^13^C labelled metabolites altered in Rv1339-expressing *M. smegmatis* mc^2^155.** The data are presented as the means±SDs from two biological replicates and three technical replicates.

|  |  | **Percentage of ^13^C labelled metabolites** | |  |
| --- | --- | --- | --- | --- |
| **Pathways** | **Metabolites** | ***M. smegmati*s pVV16** | ***M. smegmatis* pVV16::rv1339** | ***p* value** |
| Glycolysis | serine | 74.2 ± 1.9 | 80.9 ± 0.9 | 0.0015 |
| Peptidoglycan remodelling | Diaminopimelate | 66.3 ± 5.8 | 83.9 ± 0.7 | 0.0004 |
|  | lysine | 17.6 ± 2.2 | 26.9 ± 3.4 | 0.0003 |
|  | Alanyl-alanine | 50.3 ± 3.1 | 38.3 ± 5.6 | 0.0031 |
|  | *N*-acetyl-lysine | 48.9 ± 2.5 | 61.9 ± 2.0 | 4E-05 |
